# Does community-based education increase students' motivation to practice community health care? - a cross sectional study

**DOI:** 10.1186/1472-6920-11-19

**Published:** 2011-05-11

**Authors:** Masanobu Okayama, Eiji Kajii

**Affiliations:** 1 Division of Community and Family Medicine, Center for Community Medicine, Jichi Medical University, 3311-1 Shimotsuke-city Tochigi, 329-0498 Japan

## Abstract

**Background:**

Community-based education has been introduced in many medical schools around the globe, but evaluation of instructional quality has remained a critical issue. Community-based education is an approach that aims to prepare students for future professional work at the community level. Instructional quality should be measured based on a program's outcomes. However, the association between learning activities and students' attitudes is unknown. The purpose of this study was to clarify what learning activities affect students' attitudes toward community health care.

**Methods:**

From 2003 to 2009, self-administered pre- and post-questionnaire surveys were given to 693 fifth-year medical students taking a 2-week clinical clerkship. Main items measured were student attitudes, which were: "I think practicing community health care is worthwhile" ("worthwhile") and "I am confident about practicing community health care" ("confidence") using a visual analogue scale (0-100). Other items were gender, training setting, and learning activities. We analyzed the difference in attitudes before and after the clerkships by paired *t *test and the factors associated with a positive change in attitude by logistic regression analysis.

**Results:**

Six hundred forty-five students (93.1%), 494 (76.6%) male and 151(23.4%) female, completed the pre- and post-questionnaires. The VAS scores of the students' attitudes for "worthwhile" and "confidence" after the clerkship were 80.2 ± 17.4 and 57.3 ± 20.1, respectively. Both of the scores increased after the clerkship. Using multivariate logistic regression analysis, "health education" was associated with a positive change for both attitudes of "worthwhile" (adjusted RR: 1.71, 95% CI: 1.10-2.66) and "confidence" (1.56, 1.08-2.25).

**Conclusions:**

Community-based education motivates students to practice community health care. In addition, their motivation is increased by the health education activity. Participating in this activity probably produces a positive effect and improves the instructional quality of the program based on its outcomes.

## Background

In response to findings that community-based education was effective in fostering health personnel who are responsive to community needs [1,2], community-based education has been started in many medical schools around the globe as an innovative approach to medical education [3-6]. In Japan, in 2001, the Report of the Coordinating Council on the Reform of Medical and Dental Education [7] of the Ministry of Education, Culture, Sports, Science, and Technology proposed a model for an integrated medical education curriculum, i.e., a model core curriculum. All 79 Japanese medical schools were expected to implement the model core curriculum using 70% of existing contact hours to achieve their school-specific curriculum goals [8]. In 2007, the model core curriculum was revised, and it adopted a clinical training program in the community [9]. Thus, in Japan, as in Germany [10], community-based education was established as a mandatory clinical training program.

Spread of medical education into community settings has raised issues of instructional quality. Community-based clinical courses differ considerably among schools [3,4]. Japanese medical schools have similar issues surrounding community based education [11]. That is why a description of the requisite core knowledge and professional competencies are needed. Habbick and Leeder [5] summarized the rationale behind community-based education. Kristina et al [12] developed a framework to define generic objectives for it. Then, using the framework, they tried to evaluate and improve their program [13]. Coordinators who plan programs for community-based education need to identify potential areas for improvement and assure instructional quality [14].

In addition, the programs need to be evaluated from the students' viewpoint. Several studies investigating student feedback have been done [15-19]. Most of the students were satisfied with community-based education [15,16]. The community-based experience encouraged a career in general practice [17] and was positively associated with the selection of generalist residencies [18]. The community-based rural health course positively influenced many medical students to report an intention to practice in rural areas [19]. These findings were based on the students' evaluation of the program, but the students were not asked about the learning process. Thus, the findings in the previous studies were not useful for improving the quality of the program.

One of the purposes of community-based education is to encourage general physicians to practice in a community setting [1]. Thus, to improve the program, the instructional quality should be measured based not only on the students' evaluations but also on students' attitudes toward its outcomes. Although a few studies have explored the influence of learning activities in the community on students' evaluations of the program [20,21], there is no study that has examined the association between the learning activities and students' attitudes toward community health care. Knowing this association will contribute to solving the shortage of primary care physicians in the community, especially in rural areas [22]. The purpose of this study was to clarify what learning activities affect students' attitudes toward community health care.

## Methods

### Community-based clinical education in Jichi Medical University

Jichi Medical University (JMU) was established in 1972 in order to secure and improve community health care in underserved and rural areas and is managed by an education foundation that was co-founded by all 47 prefectural governments in Japan [23]. The aim of JMU is to produce doctors who practice community health care in underserved and rural areas. JMU annually recruits 2 or 3 high-school graduates from each of the 47 prefectures of Japan [24]. The students get free tuition at undergraduate institutions, and they commit to working for a medical institution in their home prefecture for 9 years after graduating from JMU.

Community-based education in JMU started with fifth-year medical students in 1998. It is a 2-week clinical clerkship in community hospitals and clinics in underserved and rural areas in their home prefectures [25].

The purposes of community-based education in JMU are the following: promote understanding of community needs and circumstances (e.g., culture, customs, social resources, and personal relationships), teach about the role of physicians practicing in a community setting, and increase motivation to work as a physician practicing community health care.

In the clinical clerkship, the students are under the supervision of 1 or 2 physician-teachers in each of the 47 prefectures. All physician-teachers graduated from JMU. They were trained in teaching skills and were appointed as regional faculty members of JMU. Although the faculty at JMU organized and managed the program and assured instructional quality, regional faculty members determined the program's process. In 2001, standards for learning activities were proposed [26], which included ambulatory care, home care, hospital care, placement in mobile clinics, on-call work, rehabilitation, health education, health check-ups, vaccination, day services, and placement in welfare facilities (welfare institutions or nursing homes for the aged). Health education was provided to the community under the supervision of the physician-teachers, public health nurses, and other health care personnel. Themes of health education are usually lifestyle-related health risks (e.g., physical inactivity, poor diet, and smoking). The students discussed health risks at the individual and community level with the participants.

### Participants and measurements

From 2003 to 2009, a self-administered questionnaire survey was given to 693 fifth-year medical students who were taking the 2-week clinical clerkship. The students were given the questionnaire before and after the clerkship. Measurement items were gender, medical institutions in clerkship, experience or not of the 11 learning activities which were ambulatory care, home care, hospital care, placement in mobile clinics, on-call work, rehabilitation, health education, health check-ups, vaccination, day services, and placement in welfare facilities, evaluation of the program, and attitudes toward community health care. The evaluation responses were: "The physician-teachers were enthusiastic," "The physician-teachers took enough time," and "The program was a worthwhile learning experience." The questions on attitudes were: "I think practicing community health care is worthwhile" ("worthwhile") and "I am confident about practicing community health care" ("confidence"). The students' evaluations and attitudes were obtained using a visual analogue scale (VAS) (0-100). The attitudes were queried in both pre- and post-questionnaire, and other measurements were done only post-questionnaire. Students identified themselves with their student identification number. This number was used to match the pre- and post-questionnaires. We assured the students that their responses would not affect their academic standing. The Bioethics Committee for Jichi Epidemiologic Research, Jichi Medical University, approved this study as exempt based on Japanese bioethical guidelines for epidemiological and clinical research proposed by the Japanese government.

### Analysis

Before statistical analyses were done, the training setting was classified as hospital, hospital and clinic, or clinic. Based on students' experience, the number of learning activities was classified into 1-4 items, 5-7 items, or 8-11 items. Based on the difference between pre- and post-questionnaire responses, we classified attitude changes as positive and non-positive. The positive-change group included the students who responded with higher VAS scores in the post-questionnaire compared to the pre-questionnaire. Statistical analyses were done using STATA/SE 11.1 for Windows (STATA Corp. LP, US). The statistical significance level was set at less than 0.05. We calculated proportion and mean ± standard deviation (SD) for the categorical data and numerical data, respectively. We analyzed the difference in students' attitudes before and after their clerkships by paired *t *test. Pearson's correlation coefficient (95% confidence interval [CI]) was used to assess the relationship between the students' evaluations of the program and the difference in students' attitudes toward community health care before and after the program. Using logistic regression analysis, crude relative ratios (RR) (95% CI) for gender, training setting, 11 learning activities, and the classification of the number of learning activities to each of the positive change groups were calculated. Then, adjusted RRs (95% CI) were obtained by adjusting for variables that were significantly related in the bivariate analyses, gender, evaluation of the program, attitudes toward community health care before the clerkship.

## Results

Of 693 students, 645 (93.1%) completed the pre- and post-questionnaires. Of these students 494 (76.6%) were male (Table 1). More than half the students trained both in hospital and clinic settings. All of them had experience in ambulatory care and 431 (66.8%) did in health education. Of the 11 learning activities recommended, the students had exposure to 7.8 ± 1.8 items (mean ± SD). The VAS scores of the students' evaluations of the physician-teachers and the program were high.

**Table 1:** Gender, training setting, learning activities, number of learning activities, and students' evaluations of the program N = 645

Gender		
Male	494	(76.6)
Female	151	(23.4)
Training setting		
Hospital	237	(36.7)
Hospital and clinic	355	(55.0)
Clinic	53	(8.2)
Learning Activities		
Ambulatroy care	645	(100.0)
Home care	580	(89.9)
Hospital care	566	(87.8)
Placement in mobile clinics	483	(74.9)
On-call work	482	(74.7)
Placement in welfare facilities*	444	(68.8)
Health education	431	(66.8)
Day services	404	(62.6)
Health check-ups	374	(58.0)
Rihabilitation	368	(57.1)
Vaccination	251	(38.9)
Number of learning activities, Mean ± SD	7.8 ± 1.8	
1-4 items	30	(4.7)
5-7 items	225	(34.9)
8-11 items	390	(60.5)
Students' evaluations for the program(VAS score, 0-100), Mean ± SD	90.1 ± 13.3	
The physician-teachers were enthusiastic.		
The physician-teachers took enough time.	88.4 ± 14.0	
The program was a worthwhile learning experience.	87.3 ± 15.6	

n(%). Abbrevation: SD, standard deviation; VAS, visual analogue scale * Welfare fasilities: welfare institution or nursing home for the aged.

The VAS scores of the students' attitudes, "worthwhile" and "confidence," after the clerkship were 80.2 ± 17.4 and 57.3 ± 20.1, respectively (Table 2). Both scores increased after the clerkship. Differences in the scores before and after the program were related to the students' evaluations of the program (Table 3).

**Table 2:** Students' attitudes toward community health care (N = 645)

	**Visual analogue scale score (0-100)**			p value*
		
	**Pre-training**	**Post-training**	**Difference (Post-Pre)**	
	
I think practicing community health care is worthwhile.	73.2 ± 18.0	80.2 ± 17.4	7.0 ± 19.0	<0.001
I am confident about practicing community health care.	50.5 ± 19.9	57.3 ± 20.1	6.8 ± 19.2	<0.001

Mean±standard deviation. *pre-training vs. post-training, paired t test

**Table 3:** Association of students' evaluations to the programme with students' attitudes toward community health care

	**"Worthwhile"**	**"Confidence"**
	
	Coefficient (95%CI)	Coefficient (95%CI)
The physician-teachers were enthusiastic.	0.247 (0.173-0318)	0.158 (0.082-0.233)
The physician-teachers took enough time.	0.155 (0.078-0.229)	0.086 (0.009-0.163)
The program was a worthwhile learning experience.	0.261 (0.188-0.332)	0.083 (0.006-0.159)

Abbrevation; Coefficient, Pearson correlation coefficient; CI, confidence interval; Worthwhile,I think practicing community health care is worthwhile; Confidence,I am confident about practicing community health care.

By univariate logistic regression analysis (Table 4), "home care" (crude RR: 1.76, 95% CI: 1.05-2.94), "health education" (1.53, 1.10-2.13), and number of learning activities (1-4 items: reference; 5-7 items: crude RR: 2.52, 95% CI: 1.16-5.49; 8-11 items: 2.27, 1.07-4.86) were associated with a positive change in the students' attitudes toward "worthwhile." Whereas "health education" (1.56, 1.11-2.17) and "health check-up" (1.48, 1.07-2.04) were associated with a positive change in students' attitudes toward "confidence." Gender and training setting were not associated with either attitude, "worthwhile" or "confidence." By multivariate logistic regression analysis, "home care" (adjusted RR: 2.04, 95% CI: 1.02-4.09) and "health education" (1.71, 1.10-2.66) were independently associated with a positive change in the students' attitudes toward "worthwhile." "Health education" (1.56, 1.08-2.25) was independently associated with a positive change in students' attitudes toward "confidence to practice."

**Table 4:** Factors associated with positive change of students' attitudes toward community health care

	**Worthwile**			**Confidence**		
	
	**Positive change†, n(%)**	**Crude RR (95%CI)**	**Adjusted RR (95%CI)‡**	**Positive change†, n(%)**	**Crude RR (95%CI)**	**Adjusted RR (95%CI)§**
	
Gender						
Male	302 (61.1)	1.00	-	297 (60.1)	1.00	-
Female	86 (57.0)	0.84 (0.58-1.22)	-	104 (68.9)	1.47 (0.99-2.17)	-
Training setting						
Hospital	142 (59.9)	1.00	-	136 (57.4)	1.00	-
Hospital and clinic	210 (59.2)	0.97 (0.69-1.35)	-	231 (65.1)	1.37 (0.98-1.94)	-
Clinic	36 (67.9)	1.42 (0.75-2.67)	-	34 (64.2)	1.33 (0.72-2.46)	-
Lerning Activities						
Ambulatroy care	388 (60.2)/-	-	-	401 (62.2)/-	-	-
Home care	357 (61.6)/31 (47.7)	1.76 (1.05-2.94)	2.06 (1.03-4.12)	357 (61.6)/44 (67.7)	0.76 (0.44-1.32)	-
Hospital care	339 (59.9)/49 (62.0)	0.91 (0.56-1.48)	-	353 (62.4)/48 (60.8)	1.07 (0.66-1.73)	-
Placement in mobile clinics	296 (61.3)/92 (56.8)	1.20 (0.84-1.73)	-	302 (62.5)/99 (61.1)	1.06 (0.74-1.53)	-
On-call work	286 (59.3)/102 (62.6)	0.87 (0.61-1.26)	-	300 (62.2)/101 (62.0)	1.01 (0.70-1.46)	-
Placement in welfare facilities*	265 (59.7)/123 (61.2)	0.94 (0.67-1.32)	-	281 (63.3)/120 (59.7)	1.16 (0.83-1.64)	-
Health education	274 (63.6)/114 (53.3)	1.53 (1.10-2.13)	1.70 (1.09-2.64)	283 (65.7)/118 (55.1)	1.56 (1.11-2.17)	1.56 (1.08-2.26)
Day services	245 (60.6)/143 (59.3)	1.06 (0.76-1.46)	-	259 (64.1)/142 (58.9)	1.25 (0.90-1.73)	-
Health check-ups	224 (60.9)/164 (59.2)	1.07 (0.78-1.47)	-	235 (63.9)/166 (59.9)	1.18 (0.86-1.63)	-
Rihabilitation	233 (62.3)/155 (57.2)	1.24 (0.90-1.70)	-	247 (90.1)/154 (56.8)	1.48 (1.07-2.04)	1.26 (0.88-1.79)
Vaccination	158 (62.9)/230 (58.4)	1.21 (0.88-1.68)	-	155 (61.8)/246 (62.4)	0.97 (0.70-1.35)	
Number of learning activities						
1-4 items	12 (40.0)	1.00	1.00	17 (56.7)	1.00	-
5-7 items	141 (62.7)	2.52 (1.16-5.49)	0.83 (0.30-2.30)	137 (60.9)	1.19 (0.55-2.57)	-
8-11 items	235 (60.3)	2.27 (1.07-4.86)	0.51 (0.18-1.50)	247 (63.3)	1.32 (0.62-2.80)	-

Abbrevation: RR, relative ratio; CI, confidence interval; Worthwhile,I think practicing community health care is worthwhile; Confidence,I am confident about practicing community health care.

* Welfare facilities: welfare institusion or nursing home for the aged.

†The number of the students with higher visual analogue scale score in the post- compared to the pre-questionnaire in each category of gender, training setting and the number of the learning acitivities and the experience/non-experience with each activity.

‡ Adjusted home care, health education, number of learning activities, gender, students' evaluations of the program, and the score of "worthwhile" before the clerkship. § Adjusted health education, health check-ups, gender, students' evaluations of the program, and the score of "confidence" before the clerkship.

## Discussion

This study clarified that students' attitudes about the importance of and confidence about practicing community health care increased after the clerkship and that the positive change was associated with the health education activity during the clerkship. Community-based education is an educational approach that aims to prepare students for future professional work at the community level [1]. In the process of behavioral change, feelings about importance and confidence contribute to the more general state of readiness to change [27]. Motivation is a person's expressed degree of readiness to change. Anything a person does to enhance his or her feelings of importance or confidence will increase his or her motivation to change [27]. Thus, just as community-based education improves attitudes toward general practice [17,18] and practicing in rural areas [19], it motivates students to practice community health care. Furthermore, our study findings indicated that the degree of readiness to change was increased by learning about community practice, and that increased motivation probably produced the positive effect of the outcome of community-based education.

 Community-based education provides students with opportunities to learn about community health problems and to apply their knowledge and skills in the provision of health services to that community [3]. In the 2-week clerkships at JMU, students provided health education to the community and discussed health risks at the individual and community level with the participants. This health education activity was associated with improved attitudes toward practicing community health care in this study. This finding indicated that the health education activity likely improved the instructional quality of the community-based education based on the outcomes. It is necessary to modify the program in community-based education to ensure that all students have access to participation in health education as the learning activity.

This study had some limitations. First, students' gender and hometown are significant factors associated with their career choices [28,29], but this study identified only gender. Gender was not associated with perceptions and attitudes toward practicing community health care in this study. Although having a rural hometown is a significant factor associated with the anticipation of practicing in a rural area [29], it is unclear how the hometown influences the anticipation of practicing community health care. Second, in community-based education in JMU, students might have learned about community involvement and interdisciplinary work in other courses. It is difficult to separate community involvement and interdisciplinary work from the health education activity. Thus, it is possible that the effect of the health education program might have included the effect of community involvement and interdisciplinary work. Third, the findings in this study are short-term effects of community-based education. More research will be needed to explore whether the effects of community-based education lead to career choices in the practice of community health care.

## Conclusions

Community-based education motivated students to practice community health care. In addition, their motivation was increased by the health education activity. Participating in this activity probably produced a positive effect and improved the instructional quality, based on outcomes.

## Competing interests

The authors declare that they have no competing interests.

## Authors' contributions

MO contributed to conception and design, acquisition of data, analysis and interpretation of data, writing and revision of the manuscript. EK contributed to conception and design and writing and revision of the manuscript. Both authors approved the final version of the manuscript to be published.

## Pre-publication history

The pre-publication history for this paper can be accessed here:

http://www.biomedcentral.com/1472-6920/11/19/prepub
